# Exploring the Relationship Between Academic Stress and Academic Engagement in Chemistry Laboratory Learning: The Mediating Role of Learning Burnout and the Differentiated Roles of Stress Sources

**DOI:** 10.3390/bs16060961

**Published:** 2026-06-10

**Authors:** Yixian Zhong, Mutong Niu, Qianfeng Zhang, Haoran Sun, Yurong Liu

**Affiliations:** School of Chemistry and Chemical Engineering, Henan Normal University, Xinxiang 453007, China; zhongyx1201@163.com (Y.Z.); niumt224@163.com (M.N.); 2025093@htu.edu.cn (Q.Z.); haoran0sun@163.com (H.S.)

**Keywords:** chemistry laboratory courses, academic stress, learning burnout, academic engagement, parallel mediation model, structural equation model

## Abstract

Academic stress is widely related to student engagement, yet its multidimensional nature and underlying processes remain insufficiently examined in laboratory learning contexts. This study explored the relationship between different sources of academic stress and academic engagement in chemistry laboratory courses, with learning burnout as a potential mediator. A cross-sectional survey was conducted among 1647 undergraduate school students. Academic stress was conceptualized as three dimensions: students’ academic self-perceptions (SP), faculty work and examinations (WE), and academic expectations (AExp). The results showed that these stress dimensions were differentially related to academic engagement. In addition, learning burnout was found to be associated with the relationship between academic stress and engagement, suggesting a mediating role. Notably, workload-related stress was more strongly related to engagement, whereas expectation-related stress showed a stronger association with burnout. These findings suggest that academic stress is not a unitary construct and that different stress sources may be associated with engagement through distinct patterns. The results provide a basis for understanding how stress operates in laboratory learning contexts and offer implications for both research and instructional practice.

## 1. Introduction

Chemistry laboratory courses are a core practical component in undergraduate chemistry education. Unlike regular theoretical courses, laboratory courses involve threefold sources of stress—operational assessments, outcome uncertainty, and multiple evaluation criteria—making them a typical context where academic stress is highly concentrated (Kyynäräinen et al., 2024). The stress that students face in laboratory courses can be categorized into three types: ongoing concerns about their own operational competence (students’ academic self-perceptions, SP), workload from lab reports and assessments (faculty work and examinations, WE), and performance pressure derived from multiple sources, including instructor expectations, parental expectations, peer comparison, and self-imposed standards (academic expectations, AExp) (Barbayannis et al., 2022). This categorization reflects a multidimensional understanding of academic stress, which is consistent with recent global evidence indicating that academic stress arises from a combination of academic, psychological, social–interpersonal, and contextual factors across educational settings (Hadi et al., 2025).

Academic engagement represents a key learning-related construct that reflects students’ active involvement in laboratory learning processes, encompassing vigor, dedication, and absorption, and has been consistently related to a range of learning-related outcomes in prior research (Schaufeli et al., 2002; Seery et al., 2024). Lower levels of engagement have been associated with reduced quality of laboratory learning and may be linked to increased risk of burnout and longer-term academic difficulties (Salmela-Aro & Upadyaya, 2014). However, whether different sources of stress are related to engagement in similar ways remains unclear.

Despite these advances, several gaps remain in the existing research. First, academic stress has often been conceptualized at a global level in prior research, although some studies have begun to recognize its multidimensional nature (Ruiz-Camacho & Gozalo, 2025; Zeng & Cong, 2025). However, the relative effect sizes and mediating proportions of SP, WE, and AExp on engagement through burnout have received limited systematic comparison. Consequently, even when educators recognize the negative implications of stress, there is limited guidance on which types of stressors may be more critical targets for intervention. Second, existing tests of mediating chains have predominantly been conducted in regular classroom contexts (Zhao et al., 2024). Whether the unique characteristics of laboratory courses—operational assessments, yield-based grading, and high outcome uncertainty—may be related to differences in these mediating relationships remains insufficiently examined (Huangfu et al., 2026).

Accordingly, this study focused on undergraduate chemistry laboratory courses as a specific context. Grounded in the dual theoretical framework of the job demands–resources (JD–R) model and conservation of resources (COR) theory, a parallel mediation model with learning burnout (LB) as the mediator was specified and examined. This study further compared the relative effect sizes and mediating proportions of three dimensions of stress—students’ academic self-perceptions (SP), faculty work and examinations (WE), and academic expectations (AExp)—on academic engagement (AE) through burnout. Additionally, exploratory multi-group SEM analysis was conducted using year level as a grouping variable to examine its potential moderating role in the mediating relationships. The findings aim to provide a more nuanced understanding of how different sources of stress are related to academic engagement in chemistry laboratory learning, thereby providing an empirical basis to inform the design of targeted interventions for distinct types of stress in chemistry laboratory instruction.

## 2. Literature Review and Research Hypotheses

### 2.1. Academic Stress and Academic Engagement in Chemistry Laboratory Learning

#### 2.1.1. Concept Definition

Academic engagement comprises three dimensions: vigor, dedication, and absorption. Vigor refers to maintaining sustained energy and the willingness to persist without giving up easily. Dedication refers to a strong sense of significance and enthusiasm invested in learning tasks. Absorption refers to the flow experience of being fully immersed in learning activities (Schaufeli et al., 2002). Academic stress in chemistry laboratory learning is defined as the subjective psychological load that students experience when they perceive that laboratory learning demands exceed their available coping resources. This stress consists of three source dimensions: SP—ongoing concerns about insufficient operational competence and knowledge base; WE—external task load from lab reports, operational assessments, and coursework; and AExp—sustained performance pressure derived from instructor expectations and self-imposed high standards (Bedewy & Gabriel, 2015). These three dimensions are independent in nature and collectively constitute the multi-source stress context of chemistry laboratory learning.

#### 2.1.2. Theoretical Mechanisms and Empirical Evidence

The energy depletion process of the job demands–resources (JD–R) model distinguishes two pathways through which academic stress impairs academic engagement (Demerouti et al., 2001): an immediate direct pathway—where learning demands directly consume the cognitive and energetic resources required for maintaining engagement; and an accumulated indirect pathway—where engagement is gradually eroded through the chronic accumulation of burnout (see Section 2.2 for details). Regarding the direct pathway, WE, characterized by high workload and time pressure, most directly consumes students’ time and energy reserves, and is therefore expected to exhibit the strongest direct effect. In contrast, AExp and SP are hypothesized to exert their influence primarily through the mediating pathway of burnout, with relatively limited direct effects.

A meta-analysis by Crawford et al. (2010), based on 259 studies, reported a negative association between hindrance learning demands and work engagement. It had an effect size of d = 0.41. This negative association remained significant even after controlling for challenge demands. Lesener et al. (2020) found, in a sample of 2356 German university students, a direct negative effect of learning demands on academic engagement (β = −0.35, *p* < 0.001). In the context of chemistry education, Naibert and Barbera (2022) found that coursework workload stress significantly and negatively predicted behavioral engagement among students in chemistry laboratory courses, indicating that WE-type stress exerts a specific inhibiting effect in this context.

Based on the above analysis, all three types of academic stress in chemistry laboratory learning can exert direct inhibiting effects on academic engagement through immediate resource consumption. Although the direct effects of AExp and SP are expected to be weaker than that of WE, the immediate resource consumption mechanism of the JD–R model suggests that all three types of stress should still exert a statistically significant direct inhibitory effect on academic engagement, albeit to varying degrees. Accordingly, the following hypothesis was proposed:

**H1.**  *The three dimensions of academic stress in chemistry laboratory learning—students’ academic self-perceptions (SP), faculty work and examinations (WE), and academic expectations (AExp)—each have a significant direct negative predictive effect on academic engagement.*

### 2.2. The Mediating Role of Learning Burnout

#### 2.2.1. Concept Definition

Burnout was initially conceptualized as a psychological syndrome associated with the occupational context (Freudenberger, 1974). As schools inherently serve as settings where students undertake “work tasks”, research on learning burnout gradually extended from the occupational domain to the educational context (Schaufeli et al., 2002). In this context, learning burnout is defined as a state of chronic exhaustion—encompassing emotional, cognitive, and behavioral dimensions—that students progressively develop under sustained high-intensity learning demands. This state comprises three components: emotional exhaustion, reduced personal accomplishment, and depersonalization (Maslach & Jackson, 1981). Chemistry laboratory courses, characterized by both high operational demands and high-stakes evaluation pressure, represent a disciplinary context where burnout is particularly prevalent.

In this study, learning burnout (LB) in chemistry laboratory learning is defined as a chronic negative psychological state that students develop as a result of persistently overloaded task demands in chemistry laboratory courses—including operational assessments, lab reports, and multiple sources of stress. This state is manifested as energy depletion, a loss of perceived meaningfulness and willingness to engage in laboratory learning, and a tendency toward behavioral withdrawal in laboratory participation.

Maslach and Leiter (1997) first proposed that burnout and engagement represent opposite poles of a continuum in academic experiences. Within this framework, Schaufeli et al. (2002) further delineated the core dimensions of these two constructs as direct opposites: emotional exhaustion was contrasted with vigor, and cynicism (depersonalization) was contrasted with dedication. Notably, this oppositional relationship reflects a theoretical connection between two related yet distinct constructs, rather than strictly representing opposite ends of a single continuum—the absence of burnout does not equate to the presence of engagement (González-Romá et al., 2006). This distinction implies that the negative prediction of engagement by burnout operates through their shared foundation of energy and motivational resources, rather than being merely a measurement-level numerical reversal (Bakker et al., 2014).

#### 2.2.2. Theoretical Relationship Between Stress, Burnout, and Engagement

The three sources of stress trigger burnout through their respective pathways of cumulative resource depletion (Hobfoll, 1989). SP erodes self-efficacy resources through repeated self-evaluations of incompetence, forming a cognitive depletion spiral. However, given the substantial individual variability in baseline self-efficacy levels, the extent to which SP translates into burnout may differ considerably across students, potentially moderating the SP → LB pathway and attenuating its average effect size at the group level. WE consumes instrumental resources through high-density task demands, though these resources are partially recoverable upon task completion. AExp chronically erodes evaluative self-resources through sustained performance standards, which cannot be fully restored by a single success within the quantitatively assessed environment of chemistry laboratories (Hobfoll, 1989). The three pathways differ in their rates of resource depletion and degrees of recoverability; accordingly, the expected effect sizes on burnout are AExp > WE > SP. Once burnout develops, emotional exhaustion depletes the energetic reserves required for vigor, reduced personal accomplishment erodes the sense of meaning on which dedication depends, and behavioral disengagement undermines the active immersion necessary for absorption. These three components collectively and systematically erode academic engagement.

#### 2.2.3. Empirical Evidence

Jagodics and Szabó (2023) found a significant positive correlation between learning demands and emotional exhaustion in a sample of 743 Hungarian undergraduate students. Lesener et al. (2020) reported a path coefficient of β = 0.38 (*p* < 0.001) from learning demands to burnout, independent of learning resource variables. A systematic review by Liu et al. (2023) indicated that this positive predictive relationship demonstrated robustness across samples varying in year level, academic discipline, and cultural context.

Schaufeli and Taris (2014) reported in their review that the correlation between burnout and engagement consistently ranged from r = −0.40 to −0.65. A longitudinal study by Salmela-Aro and Upadyaya (2014) (N = 1273) showed that burnout levels at the beginning of the semester significantly predicted changes in engagement at the end of the semester (β = −0.32, *p* < 0.001).

A meta-analysis by Crawford et al. (2010) confirmed that the negative effect of hindrance demands on engagement was substantially transmitted through emotional exhaustion. Yıldırım and Green (2024) tested a parallel mediation model examining perceived stress, multiple mediators, and subjective well-being in a university student sample. They found that indirect effects dominated the total effect, providing a methodological reference for the present study. Huangfu et al. (2026) reported a mediating chain from academic attribution to burnout to engagement in the context of chemistry learning, offering contextual evidence for the mediating role of burnout in chemistry education.

Given that the a-path effect is expected to be strongest for AExp, the proportion of its indirect effect to the total effect is also anticipated to be the highest. In contrast, WE is expected to exhibit a higher proportion of direct effect, characterized by immediate inhibition. This comparative analysis constitutes the incremental contribution of the present study relative to research that treats stress as a unitary construct. Accordingly, the following hypotheses were proposed:

**H2.**  *SP, WE, and AExp each have a significant positive predictive effect on learning burnout, with effect sizes ranked as AExp > WE > SP.*

**H3.**  *Learning burnout has a significant negative predictive effect on academic engagement.*

**H4.**  *Learning burnout plays a significant indirect mediating role in the relationships between SP, WE, AExp, and academic engagement, with the indirect effect proportion of the AExp–LB–AE pathway expected to be the highest.*

### 2.3. Theoretical Model of the Present Study

**Figure 1:**
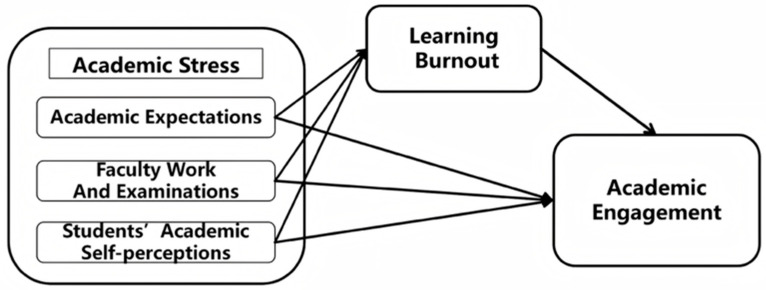
The proposed parallel mediation model of academic stress dimensions (AExp, WE, and SP), learning burnout, and academic engagement in chemistry laboratory learning.

This study constructed a parallel mediation model with learning burnout as the mediator in the context of chemistry laboratory learning (see Figure 1) to test the four formal hypotheses (H1 to H4). Building on this model, exploratory multi-group SEM analysis was conducted using year level as a grouping variable, with students divided into lower-year (freshmen and sophomores, n = 1035) and upper-year (juniors and seniors, n = 612) groups. A sequential analytical procedure was performed, including the establishment of a configural model, metric invariance testing, and structural path invariance testing with cross-group constraints (Δχ^2^ test), to examine the potential moderating direction of year level on the stress–burnout pathways.

## 3. Research Method

### 3.1. Participants

A questionnaire survey was conducted with undergraduate students majoring in chemistry-related fields—including Chemistry, Pharmaceutical Engineering, and Chemical Engineering and Technology—at a university in Henan Province, China. The sample covered all year levels from freshman to senior. All participants had completed at least one semester of chemistry laboratory courses and thus possessed basic knowledge and experiential familiarity with the instructional content and learning processes of laboratory courses.

A total of 1895 questionnaires were distributed, and 1735 were returned (response rate: 91.6%). Invalid questionnaires were excluded based on the following criteria: (1) selecting the same option for all items; and (2) responses were screened for careless responding using the LongString index, defined as the maximum number of consecutive items to which a respondent provided the same response option (Meade & Craig, 2012). A total of 88 invalid questionnaires were excluded, yielding 1647 valid questionnaires (valid response rate: 94.9%). The sample consisted of 472 males (28.7%) and 1175 females (71.3%). In terms of year level, there were 483 freshmen (29.3%), 552 sophomores (33.5%), 387 juniors (23.5%), and 225 seniors (13.7%). Regarding academic majors, 1169 students majored in Chemistry (71.0%), 219 in Chemical Engineering and Technology (13.3%), and 259 in Pharmaceutical Engineering (15.7%). Among these participants, 1035 were lower-year students (freshmen and sophomores) and 612 were upper-year students (juniors and seniors).

### 3.2. Data Collection Procedure

Online questionnaires were distributed via Wenjuanxing, a widely used online survey platform in China that supports questionnaire distribution, response collection, and data management, and has been commonly used in academic research. Student participation was voluntary and anonymous. Data collection was completed over one week in February 2026. The research protocol was approved by the Academic Ethics Committee of Henan Normal University (protocol code HNSD—2026BS—0408). Prior to data collection, all participants were informed of the research purpose and the intended use of the data. They were assured that their personal information would be kept strictly confidential and that the collected data would be used solely for academic research purposes. Participation was entirely voluntary, and no compensation or rewards were provided.

### 3.3. Measures

The questionnaire consisted of two sections: a demographic information module (including year level, gender, academic major, etc.) and a core variable measurement module. The core variables measured included Perceived Academic Stress in Chemistry Laboratory Learning (PAS), Learning Burnout in Chemistry Laboratory Learning (LB), and Academic Engagement in Chemistry Laboratory Learning (AE). Given that psychological and motivational characteristics exhibit significant domain specificity (Marsh & Craven, 2006), the research team systematically adapted each established scale to the specific context of “chemistry laboratory learning” by contextualizing all items to enhance ecological validity. The adaptation procedure was as follows: (1) general learning expressions in the original scales were replaced with chemistry laboratory operation scenarios (e.g., “during learning” was modified to “during laboratory operations”); (2) three researchers specializing in chemistry education were invited to review the adapted items and provide feedback on the accuracy of expression and disciplinary appropriateness; and (3) two undergraduate students from each major and year level were selected to participate in cognitive interviews, during which item comprehension and response experience were examined item by item, with wording subsequently refined based on the interviews. All items were rated on a 5-point Likert scale (1 = “strongly disagree,” 5 = “strongly agree”).

#### 3.3.1. Perceived Academic Stress in Chemistry Laboratory Learning (PAS)

The PAS was adapted from the Perceived Academic Stress Scale developed by Bedewy and Gabriel (2015). It was revised to specifically reflect the operational intensity, time constraints, and performance evaluation characteristics of chemistry laboratory courses. The scale comprises 18 items across three subdimensions: students’ academic self-perceptions (SP, 6 items; e.g., “I am confident that I can successfully complete experiments during laboratory sessions”); faculty work and examinations (WE, 8 items; e.g., “There is too much workload during laboratory operations”); and academic expectations (AExp, 4 items; e.g., “My instructor has unrealistic expectations of me in the laboratory”). In addition to instructor-related expectations, this dimension also captures expectation-related pressure from broader sources such as parental expectations, peer comparison, and self-imposed standards. The internal consistency of the scales was assessed using Cronbach’s alpha coefficients. In the present study, the overall reliability was α = 0.910, with subscale reliabilities of α = 0.868 (SP), α = 0.921 (WE), and α = 0.837 (AExp) (see Appendix A.1).

#### 3.3.2. Learning Burnout in Chemistry Laboratory Learning (LB)

The LB was adapted from the Maslach Burnout Inventory (MBI; Maslach & Jackson, 1981), with the application context specified to undergraduate chemistry laboratory courses. The scale comprises 20 items across three subdimensions: emotional exhaustion (EE, 8 items; e.g., “When I get up in the morning and think about today’s laboratory session, I feel exhausted”); reduced personal accomplishment (PA, 6 items; e.g., “Mastering experimental knowledge comes easily to me” reverse-scored); and depersonalization (Dep, 6 items; e.g., “I rarely preview the laboratory content in advance or organize my lab reports”). In the present study, the overall reliability was α = 0.936, with subscale reliabilities of α = 0.877 (EE), α = 0.924 (PA), and α = 0.882 (Dep) (see Appendix A.2).

#### 3.3.3. Academic Engagement in Chemistry Laboratory Learning (AE)

The AE was adapted from the Utrecht Work Engagement Scale (UWES; Schaufeli et al., 2002), with contextual optimization for the chemistry laboratory setting. The scale comprises 17 items across three subdimensions: vigor (VI, 6 items; e.g., “ During laboratory sessions, I feel full of energy”); dedication (DE, 5 items; e.g., “I feel strong and vigorous when I am conducting experiments”); and absorption (AB, 6 items; e.g., “I always persevere during experiments, even when things do not go well”). It should be noted that although learning burnout and academic engagement are conceptually distinct constructs, some item content may reflect related behavioral tendencies (e.g., reduced preparation or participation). This potential overlap is acknowledged in the interpretation of the mediation results. In the present study, the overall reliability was α = 0.937, with subscale reliabilities of α = 0.912 (VI), α = 0.906 (DE), and α = 0.926 (AB) (see Appendix A.3).

### 3.4. Data Analysis

First, descriptive statistics and correlation analyses were performed, and common method bias was assessed using Harman’s single-factor test (Podsakoff et al., 2003). However, given that all variables were measured using self-report instruments at a single time point, the potential influence of common method variance cannot be fully ruled out and should be considered when interpreting the results. Second, an exploratory factor analysis (EFA) was conducted on one randomly assigned subsample (n = 815), and a confirmatory factor analysis (CFA) was conducted on a second independent subsample (n = 832). The full sample was randomly split into two approximately equal subsamples using a randomization procedure in SPSS (v25) to ensure independence between the EFA and CFA analyses. Both subsamples exceeded commonly recommended sample size thresholds for factor analysis and structural equation modeling (e.g., minimum sample size of 200 and participant-to-item ratio greater than 10:1), supporting stable parameter estimation. The CFA was performed using Mplus 9.0 (Muthén & Muthén, 2024) with the MLR estimator. The MLR estimator was selected because it provides robust standard errors and is appropriate for Likert-type indicators with five response categories, particularly under conditions of potential non-normality. Model fit was evaluated using χ^2^/df, RMSEA, CFI, TLI, and SRMR (acceptable criteria: χ^2^/df < 5, RMSEA ≤ 0.08, CFI ≥ 0.90, TLI ≥ 0.90, SRMR ≤ 0.08; Hu & Bentler, 1999). Convergent validity was assessed using average variance extracted (AVE > 0.50) and composite reliability (CR > 0.70). Discriminant validity was established when the square root of the AVE for each factor exceeded the correlations between factors (Fornell & Larcker, 1981). Third, based on the full sample (N = 1647), a parallel mediation model with learning burnout (as a second-order factor) as the mediator was constructed. Bootstrap analysis (5000 resamples) was employed to test the three indirect pathways, with significance determined by 95% confidence intervals excluding zero. For the exploratory multi-group SEM analysis, a sequential procedure was conducted, including the establishment of a configural model, metric invariance testing (with ΔCFI ≤ 0.010 indicating invariance; Cheung & Rensvold, 2002), and structural path invariance testing with cross-group constraints (Δχ^2^ test).

## 4. Results

### 4.1. Reliability and Validity of the Measures

The EFA results (n = 815) indicated that the Kaiser–Meyer–Olkin (KMO) measure of sampling adequacy was 0.957, and Bartlett’s test of sphericity was significant (χ^2^(1485) = 31,001.987, *p* < 0.001). Principal axis factoring was employed for factor extraction, and oblique rotation (direct oblimin) was applied to account for correlations among factors. Based on the criterion of eigenvalues greater than 1, nine factors were extracted, with the cumulative variance contribution rate of the extracted loadings squared reaching 61.214%. This nine-factor structure was fully consistent with the theoretical factor structures of the PAS (three factors), the MBI (three factors), and the UWES (three factors). The pattern matrix revealed that all items loaded on their corresponding factors, with factor loadings ranging from 0.550 to 0.943, and no cross-loading items were identified (see Appendix B).

The CFA results (n = 832) demonstrated a good model fit: χ^2^(1394) = 3527.947, χ^2^/df = 2.531, RMSEA = 0.043 (90% CI [0.041, 0.045]), CFI = 0.921, TLI = 0.916, SRMR = 0.037. Regarding convergent validity, all standardized factor loadings ranged from 0.646 to 0.893 (*p* < 0.001). The average variance extracted (AVE) for each factor ranged from 0.508 to 0.666, all exceeding the recommended threshold of 0.50. Composite reliability (CR) values ranged from 0.860 to 0.919, all exceeding the recommended threshold of 0.70. Regarding discriminant validity, the square root of the AVE for each factor (ranging from 0.713 to 0.816) was greater than the correlations between that factor and any other factor (the highest absolute correlation was r = 0.523 between AE3 and PAS3), indicating that discriminant validity was satisfied.

### 4.2. Descriptive Statistics and Correlation Analysis

The Harman’s single-factor test results showed that the first unrotated factor accounted for 32.762% of the total variance, which was below the critical threshold of 40%, suggesting that common method bias was relatively limited. However, given the limited statistical power of Harman’s single-factor test, this conclusion should be interpreted with caution (Podsakoff et al., 2003).

Table 1 presents the descriptive statistics and correlation coefficients for all core variables. The mean scores for the three types of academic stress—SP, WE, and AExp—were all below the scale midpoint (ranging from 2.521 to 2.580). The mean score for learning burnout was close to the scale midpoint (M = 2.568), while the mean score for academic engagement was above the scale midpoint (M = 3.676).

Correlation analysis revealed that all three types of stress were significantly negatively correlated with academic engagement (r ranging from −0.445 to −0.565, *p* < 0.01) and significantly positively correlated with learning burnout (r ranging from 0.355 to 0.482, *p* < 0.01). Furthermore, learning burnout was significantly negatively correlated with academic engagement (r = −0.628, *p* < 0.01). These correlation patterns were consistent with the hypothesized directions of the relationships among the variables.

### 4.3. Structural Equation Modeling and Mediation Analysis

Based on the full sample (N = 1647), the structural equation model demonstrated a good overall fit: χ^2^(1414) = 5693.390, χ^2^/df = 4.026, RMSEA = 0.043 (90% CI [0.042, 0.044]), CFI = 0.928, TLI = 0.925, SRMR = 0.033. Given the relatively large sample size of this study (N = 1647), and considering that the χ^2^ statistic is highly sensitive to sample size, the model fit was evaluated comprehensively in conjunction with other fit indices, indicating an acceptable overall model fit.

The direct effect results showed that SP (β = −0.174, *p* < 0.001), WE (β = −0.253, *p* < 0.001), and AExp (β = −0.112, *p* < 0.001) were each significantly negatively related to academic engagement, supporting H1. All three types of stress had significant positive effects on learning burnout, with AExp showing the largest coefficient, followed by WE and SP (β = 0.363, *p* < 0.001) > WE (β = 0.255, *p* < 0.001) > SP (β = 0.204, *p* < 0.001), supporting H2. Learning burnout was significantly negatively related to academic engagement (β = −0.539, *p* < 0.001), supporting H3.

Bias-corrected bootstrap analysis (5000 resamples) was employed to test the three indirect pathways (see Table 2). All indirect effects had 95% confidence intervals excluding zero, indicating significant mediation effects and supporting H4. Specifically, the indirect effect of SP → LB → AE was −0.110 (95% CI [−0.145, −0.078]), accounting for 38.7% of the total effect of SP on AE (−0.284). The indirect effect of WE → LB → AE was −0.138 (95% CI [−0.179, −0.102]), accounting for 35.4% of the total effect of WE on AE (−0.390). The indirect effect of AExp → LB → AE was −0.196 (95% CI [−0.239, −0.157]), accounting for 63.6% of the total effect of AExp on AE (−0.308). All three indirect pathways were significant, and the proportion of the effect mediated by burnout was substantially higher for AExp compared to SP and WE, suggesting that the proportion of the effect mediated by burnout differed across the three types of stress.

### 4.4. Exploratory Multi-Group SEM: Potential Moderating Effect of Grade

Multi-group SEM analysis was conducted in three steps (Vandenberg & Lance, 2000). First, the configural model was established. The overall model fit met the acceptable criteria (χ^2^/df = 1.73, RMSEA = 0.030, 90% CI [0.028, 0.031], CFI = 0.929, TLI = 0.928, SRMR = 0.046), indicating that the parallel mediation structure was well-fitted for both lower-grade and upper-grade groups. Second, metric invariance was tested. After constraining factor loadings to be equal across groups, the model fit remained almost unchanged (ΔCFI = 0.000, ΔRMSEA = 0.000, Δχ^2^(10) = 12.04, *p* = 0.283), demonstrating that the measurement model achieved metric invariance across grade groups (Cheung & Rensvold, 2002). This finding justified the methodological legitimacy of comparing path coefficients across groups. Third, structural path invariance was tested with cross-group constraints (see Table 3). After constraining each stress–burnout pathway, the Δχ^2^ values were not significant for SP → LB (Δχ^2^(1) = 3.56, *p* = 0.059), WE → LB (Δχ^2^(1) = 0.01, *p* = 0.929), or AExp → LB (Δχ^2^(1) = 1.40, *p* = 0.237). These results indicated that none of the three pathways exhibited statistically significant differences across grade groups, indicating that no statistically significant differences were observed in the mediating relationships across grade groups.

Descriptive comparisons revealed that the difference in the SP → LB pathway was the largest between the two grade groups (lower grades: β = 0.281; upper grades: β = 0.178; Δ = 0.103). The grade difference in the SP → LB pathway did not reach conventional levels of statistical significance (*p* = 0.059), although a marginal trend was observed. This marginal result should be interpreted with caution, particularly given the sample size of the upper-grade subgroup and the cross-sectional nature of the data.

## 5. Discussion

### 5.1. Differential Associations of the Three Types of Stress

All three types of stress were significantly negatively related to academic engagement, with effect sizes ranked as WE (β = −0.253) > SP (β = −0.174) > AExp (β = −0.112). These findings were consistent with the predictions of the JD–R model regarding resource consumption processes (Bakker & Mostert, 2024). The strongest immediate effect was observed for WE, which aligned with the findings of Naibert and Barbera (2022) and corroborated the meta-analytic results reported by Crawford et al. (2010) (d = 0.41) as well as the findings from Lesener et al. (2020) in a university student sample (β = −0.35). Collectively, these studies indicate that high-density assessments may consume energetic resources required for engagement. In contrast, AExp exhibited the observed weakest direct effect (β = −0.112). This finding suggests that performance expectation stress may be negatively related to engagement; however, this direct relationship may not represent its primary pattern of association. Rather, AExp appears to be more strongly associated with engagement through its relationship with burnout—a point further elaborated in Section 5.3 below.

### 5.2. The Mediating Role of Learning Burnout and Structural Differences

All three types of stress significantly predicted learning burnout (H2), and learning burnout significantly predicted academic engagement (H3), collectively forming a consistent pattern of associations. Regarding the first path, the effect sizes were ranked as AExp (β = 0.363), WE (β = 0.255), and SP (β = 0.204), consistent with the predictions of H2. AExp exhibited the observed strongest effect on burnout (β = 0.363). This finding aligns with prior evidence that sustained performance expectations from instructors and self-imposed standards may reflect a sustained form of evaluative pressure. Research with university students in the Chinese educational context has demonstrated that high academic expectations are consistently associated with greater burnout accumulation (Zhang et al., 2025).

In the present study, the path coefficients from SP, WE, and AExp to learning burnout were β = 0.204, 0.255, and 0.363, respectively (all *p* < 0.001), providing direct empirical support for this relationship within the chemistry laboratory context. This finding aligns with Lesener et al. (2020), who reported a path coefficient of β = 0.38 from learning demands to burnout. SP showed the relatively observed weakest effect on burnout. This pattern may be related to individual differences in self-efficacy, which, when averaged at the group level, diluted the effect size. This finding also suggests that self-efficacy may moderate the SP–LB pathway (Chen et al., 2023), a possibility warranting further investigation in future studies. Regarding the second path, learning burnout had a significant negative effect on academic engagement (β = −0.539). This result is consistent in magnitude with the correlation range between burnout and engagement reported in previous reviews (r = −0.40 to −0.65; Schaufeli & Taris, 2014), suggesting that the negative association between burnout and engagement observed in the present study aligns with the broader literature, further corroborating the robustness of this relationship (Liu et al., 2023).

### 5.3. Two Patterns of Impairment and Implications for Intervention

The core finding of the bootstrap mediation analysis lies in the observed differences in the mediating proportions of the three indirect pathways, suggesting two distinct patterns.

Specifically, the indirect effect of AExp via burnout accounted for 63.6% of its total effect on academic engagement, substantially higher than those of WE (35.4%) and SP (38.7%). This disparity in proportions reveals not merely differences in effect sizes. For AExp, the relationship appears to be more strongly associated with burnout, with a limited direct inhibitory effect (β = −0.112). In contrast, WE and SP may be more directly related to engagement, with their direct effects (β = −0.253 and −0.174 respectively) exceeding their respective indirect effects. This distinction carries potentially direct practical implications for instructional intervention. For WE, reducing the frequency of assessments and the workload of lab reports may help to alleviate its negative association with engagement. For SP, interventions may focus on reducing students’ perceived anxiety about operational competence—for example, through pre-lab skill training and low-stakes practice sessions—so as to directly alleviate the real-time resource consumption that impairs engagement. For AExp, mitigating its negative effects requires more than simply reducing workload; it also necessitates helping students adjust their cognitive appraisals of performance expectations, such as through cognitive reappraisal and belief restructuring strategies, thereby potentially reducing the level of burnout. Yıldırım and Green (2024) observed a similar pattern in which indirect effects dominated the total effect in their parallel mediation study, providing methodological corroboration for the findings regarding the AExp pathway.

### 5.4. Implications for Research

The present study contributes to chemical education research by advancing a multidimensional understanding of academic stress within laboratory learning contexts. Rather than treating academic stress as a unitary construct, this study differentiates three distinct sources—students’ academic self-perceptions (SP), faculty work and examinations (WE), and academic expectations (AExp)—and demonstrates that these stressors are related to outcomes in partially distinct ways. This finding extends the application of the job demands–resources (JD–R) model and conservation of resources (COR) theory by highlighting heterogeneity in demand-related processes within a specific disciplinary context (Demerouti et al., 2001; Hobfoll, 1989).

In addition, the analytical framework adopted in this study provides a structured basis for future research. Researchers may build on this model by examining differentiated stress pathways using longitudinal designs to capture temporal dynamics, or by incorporating resource variables to develop a more complete JD–R framework. Furthermore, the multidimensional stress classification proposed here may be extended to other STEM laboratory settings to test its cross-disciplinary applicability.

### 5.5. Implications for Teaching

The findings of this study provide actionable implications for instructional practice in chemistry laboratory courses. Given that different sources of academic stress are associated with outcomes in different ways, targeted instructional strategies may be more effective than general stress-reduction approaches, which is consistent with the differentiated perspective on demands and resources in the JD–R framework (Demerouti et al., 2001). For workload-related stress (WE), instructors may consider reducing excessive task load or optimizing the timing and frequency of assessments to reduce perceived workload demands. For stress related to students’ academic self-perceptions (SP), providing structured skill training, scaffolded practice opportunities, and formative feedback may help reduce competence-related anxiety and enhance engagement, aligning with the importance of competence support in motivational processes (Deci et al., 2017). For academic expectation stress (AExp), instructors may focus on clarifying performance standards and supporting students in developing adaptive interpretations of expectations, thereby potentially reducing burnout levels.

Taken together, these findings suggest that aligning instructional design with specific stress mechanisms may be a promising approach to enhancing student engagement in laboratory learning contexts.

### 5.6. Implications of the Exploratory Multi-Group Analysis

The multi-group SEM results demonstrated that the stress–burnout–engagement mediating relationship was robust across lower- and upper-grade groups. This finding indicates that the JD–R energy depletion process did not show statistically significant differences across grade groups in the context of chemistry laboratory learning (Bakker & Mostert, 2024), providing cross-group support for the external validity of H1 through H4.

Nevertheless, among the three pathways, the SP → LB pathway exhibited the largest between-grade difference (lower grades: β = 0.281; upper grades: β = 0.178; Δ = 0.103). This pattern may be interpreted in light of the resource accumulation perspective of COR theory: after multiple semesters of laboratory experience, upper-grade students have accumulated relatively abundant operational self-efficacy, which may be related to lower levels of burnout associated with SP. In contrast, the between-grade difference for the WE → LB pathway was nearly negligible (Δ = 0.004), and the difference for the AExp → LB pathway was 0.044, with a slightly higher β value for upper grades, which aligns with the persistent nature of these two types of stress across grade levels. The between-grade difference in the SP → LB pathway did not reach the significance threshold (Δχ^2^(1) = 3.56, *p* = 0.059), which should be interpreted with caution of the upper-grade subsample (n = 612). Future studies are recommended to employ larger samples and longitudinal designs to formally test the moderating role of grade on the SP–LB pathway.

### 5.7. Limitations and Future Directions

Several limitations of this study should be acknowledged. First, this study employed a cross-sectional design, with all data collected at a single time point. Therefore, causal direction and temporal relationships among the variables could not be determined. Although the SEM results revealed significant mediation pathways, the current data cannot rule out the possibility that burnout precedes the perception of stress, or that the two variables mutually reinforce each other. Future research should adopt a longitudinal design, measuring SP, WE, AExp, LB, and AE at multiple time points across a semester. Such a design would capture the dynamic process of resource depletion over time and allow for a formal test of the moderating role of grade on the SP → LB pathway.

Second, all data were collected using self-report questionnaires. Although anonymous responses were employed to reduce social desirability bias and Harman’s single-factor test suggested that common method bias was relatively limited, this test has limited statistical power, and the limitations of a single-source data collection method cannot be overlooked. Future research should combine multiple data sources—such as instructor ratings, operational performance scores, or objective attendance records—to conduct triangulation and enhance the robustness of the findings.

Third, the sample was limited to undergraduate students majoring in chemistry-related fields from a single university in H Province (N = 1647), covering all four grade levels from freshman to senior. Although the sample size was relatively large and the sample encompassed diverse academic majors, and the core findings were highly consistent with the cross-context theoretical predictions of the JD–R and COR models, the conclusions of this study remain constrained by the specific institutional type and geographical scope. Future research should expand the sampling range to include institutions at different levels from various regions, as well as samples from other science and engineering laboratory courses such as physics and biology, in order to systematically test the cross-group applicability of the conclusions.

Finally, this study only included demand-side variables related to academic stress and did not incorporate JD–R resource variables such as instructor support or equipment resources, which may have underestimated the explanatory power of the overall model. Additionally, other important boundary moderators (e.g., self-efficacy) that may influence the pathways from stress to burnout and engagement were not examined. Future research should adopt a complete JD–R framework by simultaneously including both demand and resource variables, and introduce self-efficacy as a potential moderator of the SP → LB pathway, thereby constructing a more comprehensive explanatory model.

Despite these limitations, this study provides preliminary empirical evidence for the differential patterns of association of three types of stress sources in the context of chemistry laboratory learning, offering informative directions for future research and instructional intervention design in related fields.

## 6. Conclusions

Grounded in the dual theoretical framework of the job demands–resources (JD–R) model and conservation of resources (COR) theory, this study conducted a systematic empirical test of a parallel mediation model with learning burnout as the mediator in the context of chemistry laboratory learning. Based on SEM analysis of 1647 undergraduate students majoring in chemistry-related fields, all four hypotheses were supported. SP, WE, and AExp were each significantly negatively related to academic engagement (H1). All three types of stress were significantly positively related to learning burnout, with AExp showing the largest coefficient, followed by WE and SP (H2). Learning burnout was significantly negatively related to academic engagement (H3). The indirect associations via learning burnout in the relationships between the three stress dimensions and academic engagement were all significant (H4).

The core contribution of this study lies in highlighting differences in the patterns of association of the three types of stress sources. The indirect effect of AExp accounted for 63.6% of its total effect on academic engagement, substantially higher than those of WE (35.4%) and SP (38.7%). This finding suggests that academic expectations stress may be more strongly associated with engagement through its relationship with burnout, while workload stress may show a stronger direct association with engagement. This distinction extends prior research that has treated academic stress as a unitary construct, providing preliminary evidence that may inform the design of differentiated instructional approaches targeting distinct types of stress. Exploratory multi-group SEM analysis demonstrated that the mediating relationships were consistent across lower and upper grades. The descriptive difference observed in the SP → LB pathway (Δ = 0.103) may provide a basis for future longitudinal studies examining potential moderating effects.

## Figures and Tables

**Table 1:** Descriptive statistics and correlation matrix for the full sample (N = 1647).

Variable	M	SD	SP	WE	AExp	LB	AE
SP	2.521	0.618	—				
WE	2.580	0.776	0.333 **	—			
AExp	2.517	0.889	0.338 **	0.478 **	—		
LB	2.568	0.612	0.355 **	0.460 **	0.482 **	—	
AE	3.676	0.583	−0.445 **	−0.565 **	−0.515 **	−0.628 **	—

** *p* < 0.01.

**Table 2:** Path coefficients of the structural equation model and bootstrap mediation analysis results (full sample, N = 1647).

				95% Bootstrap		
Path	β	SE	*p*	LLCI	ULCI	Ratio (%)
Direct effects						
SP → AE	−0.174	0.025	<0.001	−0.224	−0.124	61.3%
WE → AE	−0.253	0.027	<0.001	−0.304	−0.200	64.9%
AExp → AE	−0.112	0.028	<0.001	−0.164	−0.058	36.4%
SP → LB	0.204	0.028	<0.001	0.149	0.257	—
WE → LB	0.255	0.031	<0.001	0.195	0.317	—
AExp → LB	0.363	0.028	<0.001	0.308	0.421	—
LB → AE	−0.539	0.035	<0.001	−0.606	−0.471	—
Indirect effects						
SP → LB → AE	−0.110	0.017	<0.001	−0.145	−0.078	38.7%
WE → LB → AE	−0.138	0.019	<0.001	−0.179	−0.102	35.4%
AExp → LB → AE	−0.196	0.021	<0.001	−0.239	−0.157	63.6%
Total effects						
SP	−0.284	0.026	<0.001	−0.334	−0.233	100%
WE	−0.390	0.026	<0.001	−0.441	−0.339	100%
AExp	−0.308	0.025	<0.001	−0.356	−0.259	100%

**Table 3:** Results of cross-group comparisons of structural paths in exploratory multi-group SEM.

Path	Lower Grade β	Upper Grade β	Δβ	Δχ^2^(1)	*p*
SP → LB	0.281	0.178	0.103	3.56	0.059
WE → LB	0.332	0.336	0.004	0.01	0.929
AExp → LB	0.352	0.396	0.044	1.40	0.237

## Data Availability

The data presented in this study are available on request from the corresponding author.

## Abbreviations

The following abbreviations are used in this manuscript:

PAS	Perceived Academic Stress in Chemistry Laboratory Learning
LB	Learning Burnout in Chemistry Laboratory Learning
AE	Academic Engagement in Chemistry Laboratory Learning
SP	Students’ academic self-perception
WE	Faculty work and examinations
AExp	Academic expectations
EE	Emotional exhaustion
Dep	Depersonalization
PA	Reduced personal accomplishment
VI	Vigor
DE	Dedication
AB	Absorption

## Appendix A. Complete Adapted Measurement Instruments Used in This Study

The following scales were contextually adapted based on established instruments and anchored to the specific context of “chemistry laboratory learning” (see Section 3.3 for the detailed adaptation process: expert review + cognitive interviews). All items were rated on a 5-point Likert scale (1 = strongly disagree; 5 = strongly agree). Reverse-scored items are marked with an asterisk (*) and should be reverse-coded prior to analysis (new score = 6 − original score). The subdimension groupings are consistent with those described in the main text.

### Appendix A.1. Perceived Academic Stress in Chemistry Laboratory Learning Scale (PAS; 18 Items)

Adapted from Bedewy and Gabriel (2015). This scale comprises three subdimensions: students’ academic self-perceptions (SP, 6 items, with Items 1, 2, and 3 being reverse-scored), faculty work and examinations (WE, 8 items, with Items 4 and 5 being reverse-scored), and academic expectations (AExp, 4 items).

**Section Number**	**Question**
1 *	I am confident that I can successfully complete experiments during laboratory sessions.
2 *	I believe that the abilities cultivated through chemistry laboratory courses are important for my future career development.
3 *	When conducting experiments, I can easily make decisions when faced with situations requiring judgment or adjustment.
4 *	The time I allocate to laboratory sessions and lab assignments is sufficient.
5 *	I have enough time to relax after completing laboratory sessions.
6	My instructor is not very satisfied with my laboratory performance.
7	I am afraid of failing this year’s laboratory course.
8	I think my worry about laboratory exams is a personal flaw.
9	My instructor has unrealistic expectations of me in the laboratory.
10	There is too much workload during laboratory operations.
11	I think the amount of homework assigned for laboratory courses is too heavy.
12	If I fall behind in the laboratory sessions, I will not be able to catch up.
13	My parents’ unrealistic expectations cause me a lot of stress.
14	The competition with my classmates over laboratory grades is quite intense.
15	The questions on laboratory exams are usually very difficult.
16	The time for laboratory exams is too short for me to complete all the questions.
17	During laboratory exams, I always feel so stressed that I cannot bear it.
18	Even if I pass the laboratory exams, I worry that I will not find a job.
* Reverse-scored items.	

### Appendix A.2. Learning Burnout in Chemistry Laboratory Learning Scale (LB; 20 Items)

Adapted from the Maslach Burnout Inventory (Maslach & Jackson, 1981). This scale comprises three subdimensions: emotional exhaustion (EE, 8 items, all positively scored), reduced personal accomplishment (6 items, all reverse-scored), and depersonalization (Dep, 6 items, with Items 1 and 8 being reverse-scored).

**Section Number**	**Question**
1 *	I can understand the experimental principles and independently complete the given laboratory operations.
2	I feel that the experimental knowledge I have learned is completely useless.
3 *	Mastering experimental knowledge comes easily to me.
4	When I get up in the morning and think about today’s laboratory session, I feel exhausted.
5	I find it difficult to sustain lasting enthusiasm for experiments.
6 *	During experiments, I can handle my emotional issues calmly.
7	I feel drained after completing laboratory sessions.
8 *	So far, university laboratory courses have fully allowed me to demonstrate my abilities.
9	I feel bored with experiments.
10	I rarely study experiment-related content after laboratory sessions.
11 *	I feel competent in university laboratory courses.
12	I often doze off during laboratory sessions.
13 *	I am interested in the experiments I conduct.
14	I feel that I lack sufficient patience during laboratory work.
15 *	For me, completing laboratory courses and achieving good grades is easy.
16	I only preview or review experimental content when lab reports are due or before exams.
17	I want to do experiments but find them tedious.
18 *	I feel energetic during experiments.
19	I rarely preview laboratory content in advance or organize my lab reports.
20	Laboratory exams always bore me.
* Reverse-scored items.	

### Appendix A.3. Academic Engagement in Chemistry Laboratory Learning Scale (AE; 17 Items)

Adapted from the Utrecht Work Engagement Scale (Schaufeli et al., 2002). This scale comprises three subdimensions: vigor (6 items), dedication (5 items), and absorption (6 items).

**Section Number**	**Question**
1	During laboratory sessions, I feel full of energy.
2	I feel strong and vigorous when I am conducting experiments.
3	When I get up in the morning, I feel like going to the laboratory session.
4	I can continue conducting experiments for very long periods without needing a break.
5	Even when I feel mentally fatigued, I can recover quickly during experiments.
6	I always persevere during experiments, even when things do not go well.
7	I find the experiments that I do full of meaning and purpose.
8	I am enthusiastic about conducting experiments.
9	My experiments inspire me.
10	I am proud of the experiments that I do.
11	To me, my experiments are challenging.
12	When I am conducting experiments, time flies.
13	When I am conducting experiments, I forget everything else around me.
14	I feel happy when I am fully immersed in experiments.
15	I can get carried away by my experiments.
16	I am immersed in my experiments.
17	Once I start experimenting, it is difficult for me to detach from it.
Scoring Instructions: (1) Mean scores or total scores can be calculated for each subdimension. (2) Reverse-scored items should be reverse-coded prior to calculating mean scores.	

## Appendix B. Detailed Psychometric Properties of the Adapted Scales

### Pattern Matrix of the Exploratory Factor Analysis (EFA)

**Item**	**Factor**	**SP**	**WE**	**AExp**	**EE**	**Dep**	**PA**	**VI**	**DE**	**AB**
PAS01	**SP**	**0.726**	−0.080	−0.022	−0.033	0.057	0.045	−0.012	0.006	−0.012
PAS02		**0.758**	−0.036	−0.078	−0.079	0.046	0.067	−0.032	0.016	0.027
PAS03		**0.817**	0.038	−0.025	0.012	−0.038	0.051	0.010	−0.029	0.005
PAS07		**0.746**	−0.001	0.041	0.060	−0.016	−0.075	−0.024	0.034	−0.017
PAS08		**0.682**	0.052	0.052	0.034	−0.031	−0.089	0.070	−0.023	−0.010
PAS18		**0.620**	0.023	0.058	0.049	−0.067	−0.019	−0.031	0.011	−0.065
PAS04	**WE**	0.051	**0.735**	−0.145	−0.057	0.009	0.025	−0.056	−0.061	0.064
PAS05		−0.020	**0.836**	−0.087	−0.016	−0.055	0.014	−0.004	−0.060	0.019
PAS10		0.021	**0.661**	0.034	−0.048	0.072	0.003	−0.049	−0.014	−0.013
PAS11		−0.070	**0.795**	0.013	−0.007	0.002	−0.020	−0.027	0.010	−0.067
PAS12		0.025	**0.853**	0.048	0.018	−0.006	−0.005	0.022	0.031	0.033
PAS15		−0.023	**0.786**	0.056	0.044	−0.025	−0.010	0.027	0.035	−0.026
PAS16		−0.032	**0.835**	0.034	0.054	−0.003	−0.020	0.018	0.034	−0.039
PAS17		0.055	**0.593**	0.057	−0.008	0.071	0.063	0.033	−0.010	−0.008
PAS06	**AExp**	0.079	−0.051	**0.588**	−0.068	0.049	0.065	0.036	−0.081	0.011
PAS09		−0.020	−0.002	**0.811**	0.042	0.017	0.009	−0.066	0.054	0.049
PAS13		−0.024	0.045	**0.837**	−0.038	−0.004	−0.012	0.013	−0.034	0.002
PAS14		−0.006	−0.021	**0.717**	0.043	−0.043	−0.007	−0.024	0.005	−0.051
LB03	**EE**	0.004	0.032	−0.041	**0.679**	−0.092	0.058	−0.080	−0.016	0.001
LB06		−0.040	0.006	−0.031	**0.550**	0.012	0.052	−0.044	−0.020	−0.003
LB11		−0.031	0.020	0.050	**0.727**	−0.018	0.070	0.073	0.013	−0.008
LB13		−0.019	−0.039	−0.005	**0.822**	0.102	−0.050	0.035	−0.021	−0.007
LB15		0.082	0.030	0.047	**0.741**	−0.061	−0.021	0.005	−0.012	0.017
LB18		0.031	−0.051	−0.034	**0.816**	0.125	−0.036	−0.012	0.027	0.037
LB02	**Dep**	−0.031	0.047	0.045	−0.036	**0.761**	−0.091	0.011	0.055	−0.003
LB04		0.010	0.010	−0.029	−0.018	**0.810**	−0.030	0.021	−0.028	0.007
LB05		0.029	0.019	−0.074	0.018	**0.750**	−0.001	−0.038	−0.032	0.000
LB07		0.084	−0.013	0.012	−0.044	**0.618**	0.097	0.002	−0.066	0.061
LB09		−0.031	−0.029	0.031	0.030	**0.853**	0.001	0.005	0.025	−0.048
LB12		−0.042	−0.008	0.032	0.062	**0.805**	0.005	0.023	−0.015	0.009
LB17		0.021	0.017	−0.011	0.045	**0.738**	0.039	−0.010	0.018	−0.017
LB20		−0.049	−0.046	−0.016	0.004	**0.765**	0.025	−0.056	−0.011	−0.071
LB01	**PA**	−0.080	−0.021	−0.078	0.143	−0.146	**0.679**	−0.080	−0.026	−0.086
LB08		−0.052	−0.037	−0.007	0.074	−0.100	**0.943**	0.010	−0.011	−0.054
LB10		0.037	−0.016	0.022	0.006	0.137	**0.686**	−0.020	0.020	0.025
LB14		0.071	0.085	0.005	−0.017	0.085	**0.574**	0.070	0.024	0.031
LB16		0.029	0.003	0.065	−0.097	0.021	**0.775**	0.025	0.025	0.034
LB19		−0.002	0.051	0.028	0.004	0.153	**0.666**	0.008	0.007	0.047
AE01	**VI**	0.003	−0.027	0.053	−0.009	−0.006	−0.019	**0.800**	−0.002	−0.011
AE04		−0.017	0.030	0.005	0.030	−0.022	0.031	**0.878**	0.008	0.032
AE08		0.003	−0.049	−0.016	−0.016	−0.062	−0.018	**0.722**	−0.032	−0.057
AE12		−0.034	−0.072	0.025	−0.028	−0.017	0.014	**0.709**	0.008	−0.030
AE15		0.004	−0.007	−0.043	0.013	0.025	−0.004	**0.839**	0.009	−0.008
AE17		0.024	0.065	−0.075	−0.007	0.044	0.016	**0.761**	0.023	0.079
AE02	**DE**	−0.009	−0.020	0.036	−0.011	0.020	−0.019	0.035	**0.802**	−0.009
AE05		0.024	0.027	0.007	0.033	−0.047	−0.008	0.000	**0.889**	0.015
AE07		0.012	0.013	−0.058	0.027	0.003	−0.003	0.023	**0.815**	−0.002
AE10		−0.040	−0.009	−0.017	−0.045	0.014	0.046	−0.025	**0.826**	−0.006
AE13		0.031	−0.077	−0.008	−0.042	−0.027	0.018	−0.012	**0.641**	0.025
AE03	**AB**	−0.022	0.009	−0.008	0.005	0.075	−0.088	0.025	0.066	**0.723**
AE06		0.006	−0.015	−0.013	0.035	−0.052	−0.042	−0.002	−0.050	**0.789**
AE09		0.006	0.026	−0.010	−0.018	0.013	0.022	−0.029	0.061	**0.867**
AE11		−0.012	0.014	0.011	0.029	−0.023	−0.012	−0.008	−0.004	**0.882**
AE14		−0.010	0.005	0.016	0.003	−0.023	0.034	0.009	−0.022	**0.879**
AE16		−0.028	−0.077	0.016	−0.026	−0.045	0.059	0.017	−0.032	**0.719**
Note: KMO = 0.957; Bartlett’s test of sphericity: χ^2^(1485) = 31,001.987, *p* < 0.001. Nine factors were extracted, with a cumulative variance contribution rate of 61.214%. Extraction method: principal axis factoring. Rotation method: oblimin with Kaiser normalization (converged after 7 iterations). Factor loadings ≥0.40 are shown in bold.										
